# Design of pH/Redox Co-Triggered Degradable Diselenide-Containing Polyprodrug via a Facile One-Pot Two-Step Approach for Tumor-Specific Chemotherapy

**DOI:** 10.3390/molecules29163837

**Published:** 2024-08-13

**Authors:** Yanru Hu, Peng Liu

**Affiliations:** State Key Laboratory of Applied Organic Chemistry, College of Chemistry and Chemical Engineering, Lanzhou University, Lanzhou 730000, China; huyr20@lzu.edu.cn

**Keywords:** tumor chemotherapy, drug self-delivery system, polyprodrug, pH/redox co-triggered degradation, diselenide bond

## Abstract

The diselenide bond has attracted intense interest for drug delivery systems (DDSs) for tumor chemotherapy, owing to it possessing higher redox sensitivity than the disulfide one. Various redox-responsive diselenide-containing carriers have been developed for chemotherapeutics delivery. However, the premature drug leakage from these DDSs was significant enough to cause toxic side effects on normal cells. Here, a pH/redox co-triggered degradable polyprodrug was designed as a drug self-delivery system (DSDS) by incorporating drug molecules as structural units in the polymer main chains, using a facile one-pot two-step approach. The proposed PDOX could only degrade and release drugs by breaking both the neighboring acid-labile acylhydrazone and the redox-cleavable diselenide conjugations in the drug’s structural units, triggered by the higher acidity and glutathione (GSH) or reactive oxygen species (ROS) levels in the tumor cells. Therefore, a slow solubility-controlled drug release was achieved for tumor-specific chemotherapy, indicating promising potential as a safe and efficient long-acting DSDS for future tumor treatment.

## 1. Introduction

Owing to the higher GSH and ROS levels in tumor cells than in normal cells, redox-responsive DDSs have been widely investigated as tumor-targeting nanomedicines in recent decades [1,2] in order to improve antitumor efficacy and suppress the toxic side effects of chemotherapeutics on normal cells. Moreover, the most widely used redox linker, the disulfide bond, has been used to conjugate drugs onto the carriers to form redox-triggered prodrugs [3,4], for a better controlled drug release with minimized premature drug leakage from the DDSs via non-covalent drug-loading.

Polyprodrugs [5,6,7], in which the drug molecules are incorporated as structural units in the polymer main chains, were developed as DSDSs. Owing to the two dynamic covalent conjugations neighboring the drug unit, a more precise tumor-selective controlled-drug-release performance could be achieved, in comparison with the conventional polymer prodrugs with only one conjugation. Moreover, multi-triggered drug release could be designed by combining different stimulus-responsive conjugations [8,9,10,11].

Like the disulfide bond, the diselenide bond could also be cleaved by reduction with GSH or oxidation with ROS. Moreover, it possesses a higher redox sensitivity than the disulfide bond [12]. Recently, it has been widely used in redox-triggered DDSs for chemotherapeutics delivery, by designing the diselenide-containing carriers for drug encapsulation [13,14,15,16,17,18]. Up to now, there has been very little work carried out on the polymer prodrug concerning the conjugation of drugs onto the polymer via the diselenide bond [19].

Besides redox-cleavage, the diselenide bond could also be cleaved by X-ray [20,21], γ-ray [22] and near-infrared (NIR) laser [23,24]. This means that the endogenous stimuli-triggered drug release from the diselenide-containing nanomedicines could also be accelerated by exogenous irradiation, meaning a more regulable drug release performance. Moreover, the selenide-containing polymers were found to selectively induce cancer cells to express excessive ROS, thus causing significant cellular apoptosis as a drug-free therapeutic system [25,26,27].

**Scheme 1:**
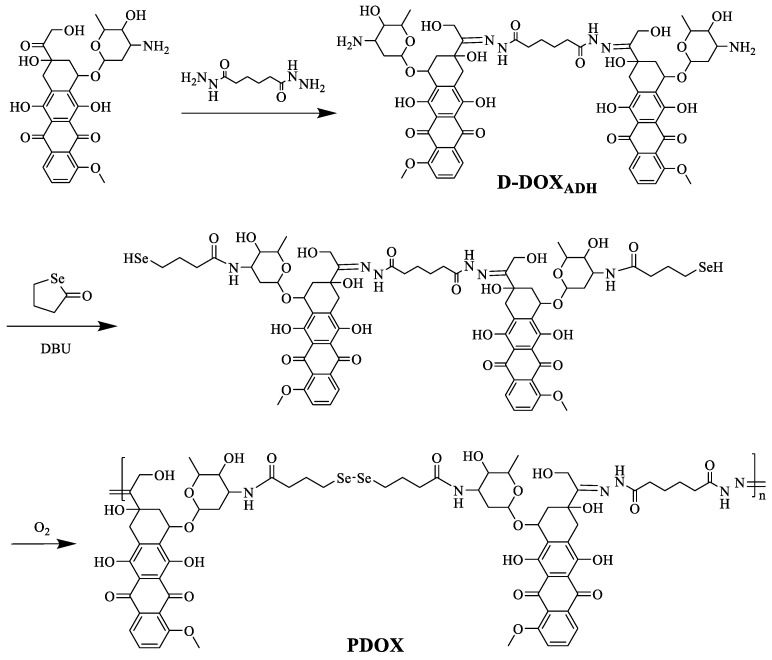
Synthesis of the pH/redox co-triggered degradable polyprodrug (PDOX).

Based on the unique advantages of the diselenide bond, the pH/redox co-triggered degradable polyprodrug was designed as a drug self-delivery system (DSDS) by incorporating drug molecules as structural units into the polymer main chains, by a facile one-pot two-step reaction, with selenolactone and an acid-labile dimer of doxorubicin (D-DOX_ADH_) (Scheme 1). It provided a slow, sustained drug release triggered by endogenous stimuli such as higher acidity and GSH or ROS levels in the tumor intracellular microenvironment, due to the solubility of the released derivatives selenol (DOX-SeH) or seleninic acid (DOX-SeOOH), respectively.

## 2. Results

### 2.1. Synthesis and Characterization of PDOX

**Scheme 2:**
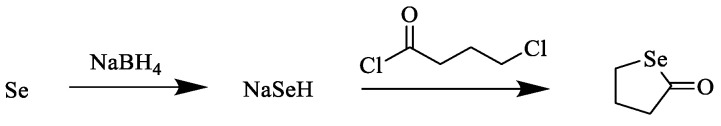
Synthesis of selenolactone.

**Figure 1:**
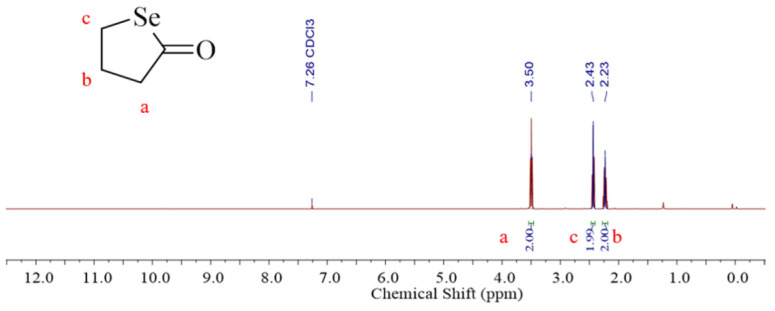
^1^H NMR spectrum of selenolactone.

**Figure 2:**
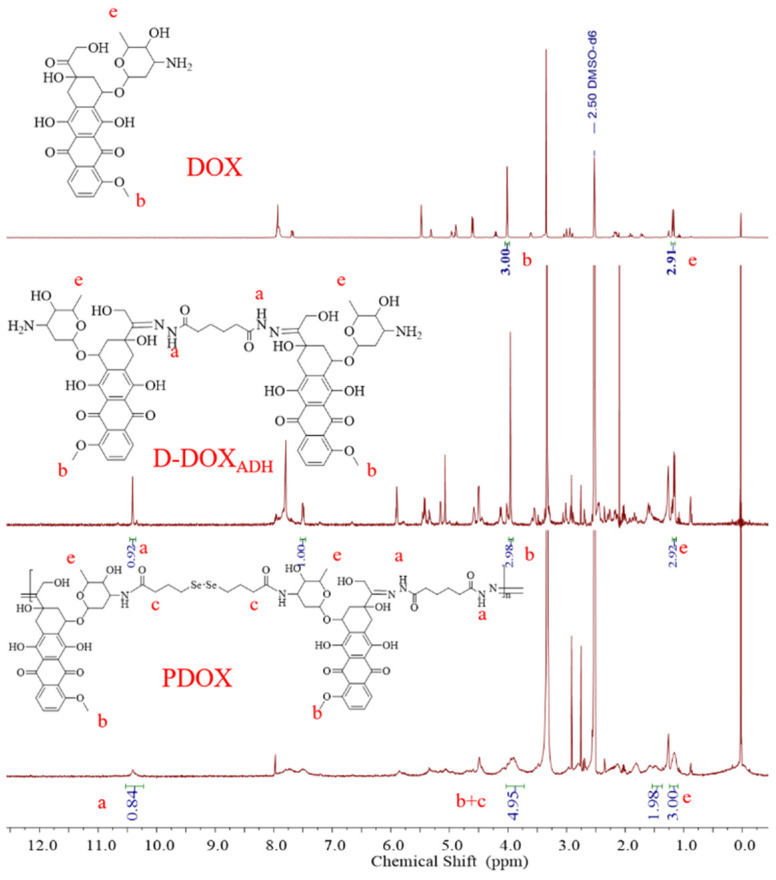
^1^H NMR spectra of DOX, D-DOX_ADH_ and PDOX.

Selenolactone was synthesized with selenide (Se), NaBH_4_ and 4-chlorobutyryl chloride according to the reported work (Scheme 2) [28], with a yield of 47.67% and revealed by ^1^H NMR analysis ((400 MHz, chloroform-d): δ = 3.50 ppm (d, 2.00H), δ = 2.43 ppm (d, 1.99H), δ = 2.23 ppm (t, 2.00H)) (Figure 1). D-DOX_ADH_ was synthesized by conjugating DOX with adipic dihydrazone (ADH) according to the reported work [29], with a yield of 78.80% and revealed by ^1^H NMR ((400 MHz, DMSO-*d*_6_): δ = 10.39 ppm, 0.93H, δ = 7.96–7.82 ppm, 2.06H, δ = 7.50–7.45 ppm, 1.00H, δ = 3.95–3.90 ppm, 3.03H)) (Figure 2).

**Figure 3:**
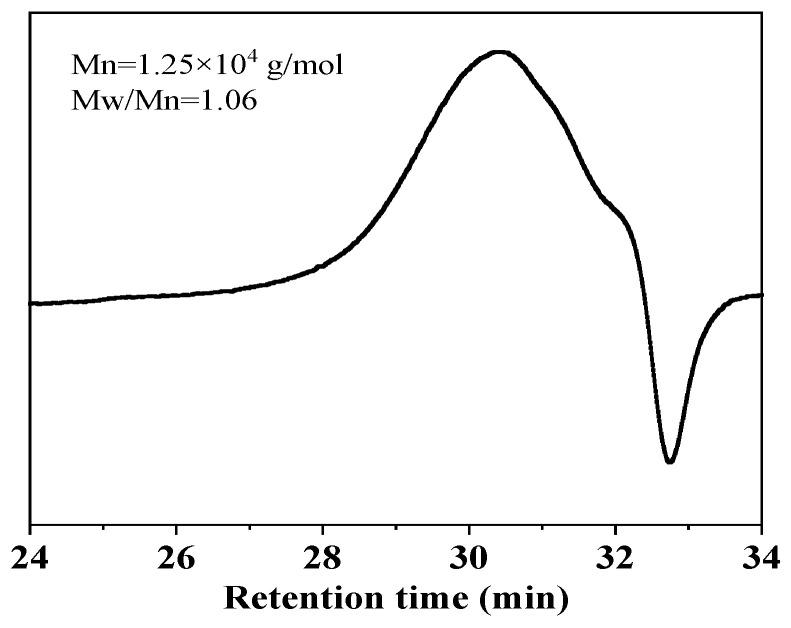
GPC curve of the proposed PDOX.

In the polymerization, selenolactone was ring-opened with the two amino groups on the D-DOX_ADH_ producing the intermediate (selenol derivative of D-DOX_ADH_). Because the reaction was conducted in air, the selenol derivative could be easily coupled by oxidation with O_2_ in air. Thus, the proposed pH/redox co-triggered degradable polyprodrug (PDOX) was obtained. D-DOX_ADH_ is soluble in both DMF and ethanol; a red precipitate was obtained after adding ethanol to the resultant solution, meaning successful stepwise polymerization via ring-opening/oxidation. The red precipitate was analyzed using the GPC technique; a number-average molecular weight of 1.25 × 10^4^ was achieved with a polydispersity index (PDI) of 1.06 (Figure 3).

In the ^1^H NMR spectrum (Figure 2), the signal of the protons adjacent to the carbonyl group was overlapped with that of the methoxy group on DOX at δ = 3.97–3.72 ppm. The protons in the amide group on D-DOX_ADH_ and the methyl group on DOX showed the signals at the chemical shifts of δ = 10.49–10.20 ppm and δ = 1.13–1.01 ppm, respectively. The integral area of the three signals was 4.95:0.84:3.00. It was near to the theoretical value of 5:1:3, indicating the successful synthesis of the proposed PDOX via the open-ring reaction and oxidation coupling reaction in one pot (Scheme 1).

Furthermore, the DOX content of the proposed PDOX was determined as 1.30 × 10^−3^ mmol/g by measuring the absorption at 480 nm of its DMSO solution with UV–vis spectrometer and calculating with the calibration curve of DOX in DMSO. It was very near to the theoretical value of its repeating unit (1.31 × 10^−3^ mmol/g), also revealing the successful synthesis of the proposed PDOX.

### 2.2. Fabrication and Characterization of PDOX Nanoparticles

The PDOX nanoparticles were fabricated by dialyzing the PDOX solution in DMSO with different concentrations against water (molecular weight cutoff (MWCO) of 1000). During the dialysis, the good solvent of PDOX (DMSO) diffused out of the dialysis bag, while the bad solvent of PDOX (water) diffused into the dialysis bag. Therefore, the PDOX solution in the dialysis bag suffered a transformation from good solvent to bad solvent. Therefore, the PDOX was slowly fabricated due to the π–π stacking interaction between the anthracene rings in the DOX units [30].

**Figure 4:**
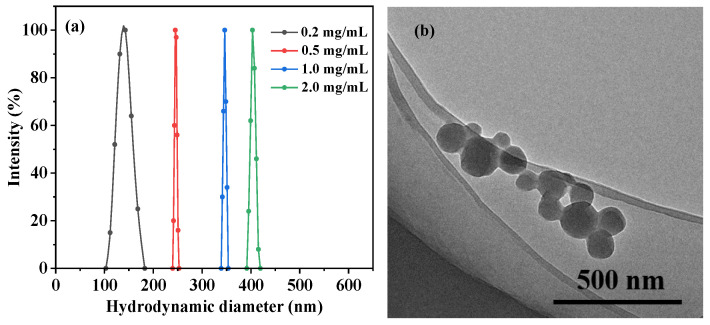
Typical hydrodynamic diameter and distribution of the PDOX nanoparticles by dialysis at different concentrations (**a**) and the TEM image of the PDOX nanoparticles fabricated at 0.2 mg/mL (**b**).

Increasing the PDOX concentration from 0.20 mg/mL to 2.0 mg/mL, the mean hydrodynamic diameter (Dh) of the resultant PDOX nanoparticles increased significantly from 143 nm (PDI of 0.199) to 245 nm (PDI of 0.018), 346 nm (PDI of 0.015) and 403 nm (PDI of 0.024), respectively (Figure 4a). For a better passive targeting via the enhanced permeability and retention (EPR) effect, the nanoparticles fabricated at a PDOX concentration of 0.2 mg/mL were selected for further investigation. In the TEM observation (Figure 4b), the PDOX nanoparticles showed a near spherical shape with a particle size in the range of 75–230 nm. Its mean particle size was approximately 107 nm, smaller than the Dh from the DLS analysis, due to its swelling and/or surface hydration.

### 2.3. In Vitro Drug Release

**Figure 5:**
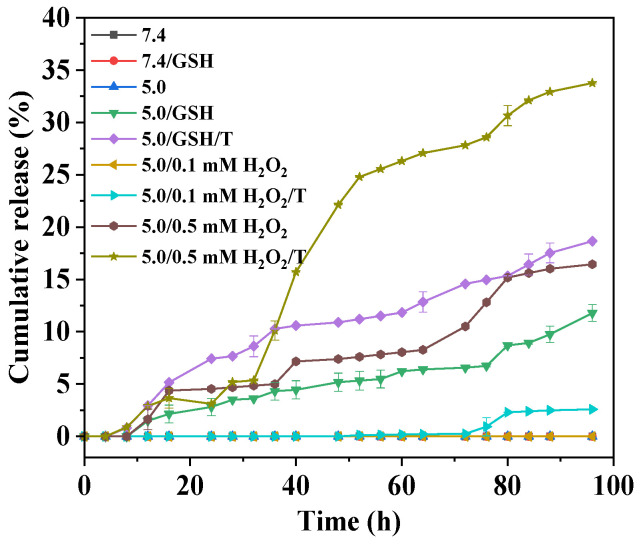
Drug release profiles of the PDOX nanoparticles in different media.

The pH/redox co-triggered degradation and drug release from the proposed PDOX nanoparticles were investigated in vitro in different media (Figure 5). Clearly, there was no obvious drug leakage in the weak basic media, even with a high GSH level of 10 mM (7.4/GSH). It ensured the safety of the proposed polyprodrug-based nanomedicine, efficiently avoiding premature drug leakage into the blood circulation.

In the acidic media mimicking the intracellular microenvironment, the drug could not be released with any GSH or H_2_O_2_ (5.0), and the cumulative release was only 12% and 16% with 10 mM GSH (5.0/GSH) or 0.5 mM H_2_O_2_ (5.0/0.5 mM H_2_O_2_) in 96 h, respectively. And no obvious drug release was observed in the media with 0.1 mM H_2_O_2_ (5.0/0.1 mM H_2_O_2_). Such a feature revealed the excellent pH/redox co-triggered degradation and drug release from the proposed PDOX nanoparticles, also avoiding drug mis-release into normal cells with the same acidity but much lower GSH and H_2_O_2_ levels.

Moreover, the drug release was accelerated distinctly by adding surfactant (0.1% Tween-80) in the releasing media. The cumulative release increased to 19% and 34% in the acidic media with high GSH (5.0/GSH/T) and H_2_O_2_ (5.0/0.5 mM H_2_O_2_/T) levels. These phenomena demonstrated that the released drug possessed a lower solubility in aqueous media. Based on the results, it was deduced that the DOX derivatives selenol (DOX-SeH) and seleninic acid (DOX-SeOOH) were released by cleaving the diselenide bond in the polyprodrug via reduction with GSH or oxidation with H_2_O_2_, respectively [31,32]. Moreover, the cumulative release was higher with a high H_2_O_2_ level than with a high GSH level, regardless of Tween-80, because of the more hydrophilic qualities of derivative seleninic acid than of selenol. The results indicated a solubility-controlled drug release performance by the pH/redox co-triggered polyprodrug.

**Scheme 3:**
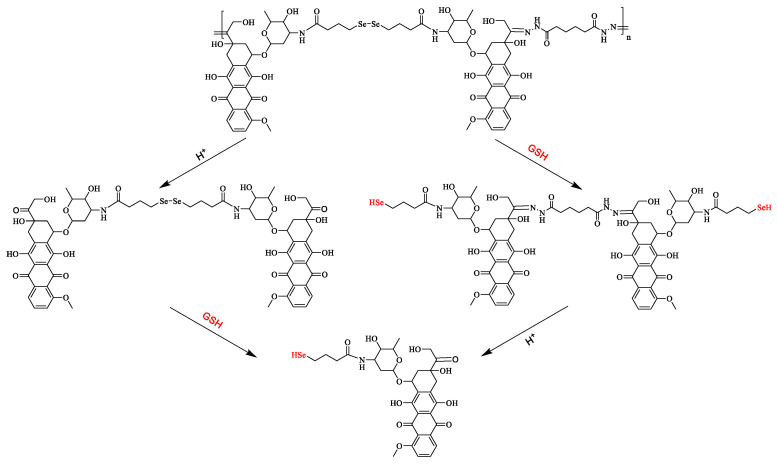
pH/Reduction co-triggered degradation of PDOX to release the selenol (DOX-SeH).

**Scheme 4:**
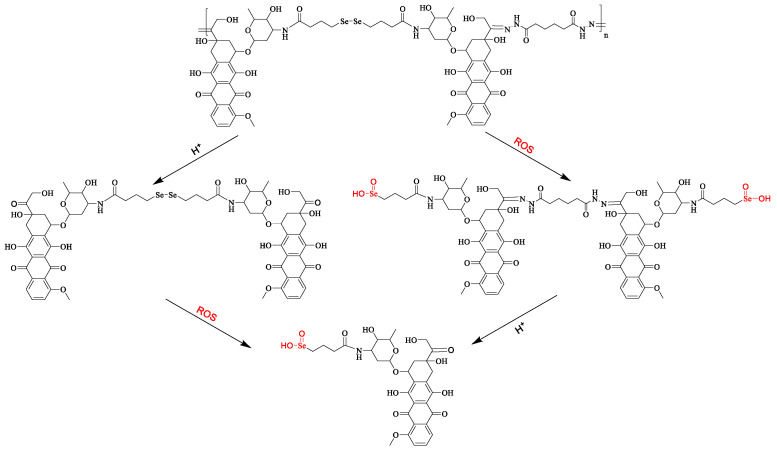
pH/Oxidation co-triggered degradation of PDOX to release the seleninic acid (DOX-SeOOH).

On the other hand, obvious accelerated drug release stages could be seen in the acidic media with high GSH or H_2_O_2_ level. And the acceleration occurred earlier in the presence of Tween-80. Due to the hydrophobic nature of the polyprodrug as well as the π–π stacking interaction between the anthracene ring in the DOX units, the stimuli (H^+^ and GSH or H_2_O_2_) could hardly enter the PDOX nanoparticles, but only act on their surfaces. The surface polyprodrug blocks were cleaved into oligomers, then dimers and finally the DOX derivatives selenol (DOX-SeH) (Scheme 3) and seleninic acid (DOX-SeOOH) (Scheme 4). Due to the cleavage of the diselenide bond and release, the polarity of the oligomers and dimers increased, favoring the attack of the stimuli, leading to an acceleration. Owing to the higher polarity and hydrophilicity of the seleninic acid (DOX-SeOOH), faster drug release was achieved with a high H_2_O_2_ level than a high GSH level.

In any case, the solubility of the DOX derivatives selenol (DOX-SeH) and seleninic acid (DOX-SeOOH) is low, and the oligomers and dimers could barely be dissolved in water. With the help of Tween-80, more DOX derivatives were dissolved in the releasing media. On the other hand, Tween-80 would also interact with the oligomers and dimers in the surface layer, also enhancing hydrophilicity and subsequently enabling the entry and attack of the stimuli.

### 2.4. Cellular Uptake and Cytotoxicity

**Figure 6:**
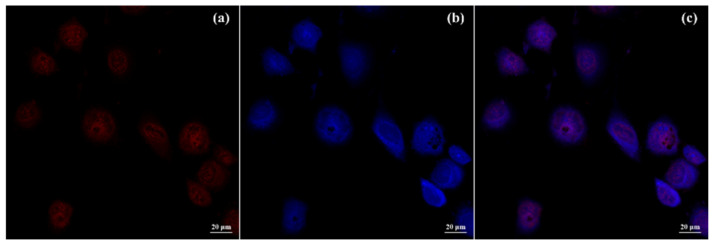
CLSM images of the HepG2 cells after incubation with the PDOX nanoparticles (15 μg/mL) for 48 h: DOX (**a**), DAPI (**b**) and merged (**c**) (scale bar: 20 μm).

The successful cellular uptake was revealed by the CLSM analysis of the human cancer cells (HepG2) after co-incubation with the proposed PDOX nanoparticles at a dosage of 15 μg/mL for 48 h (Figure 6). The red fluorescence of DOX overlapped perfectly with the blue fluorescence of the DAPI-stained nuclei, indicating the internalized PDOX nanoparticles were triggered to degrade and release the DOX derivatives, selenol (DOX-SeH) and seleninic acid (DOX-SeOOH), in the tumor intracellular microenvironment with high acidity and GSH/ROS levels.

**Figure 7:**
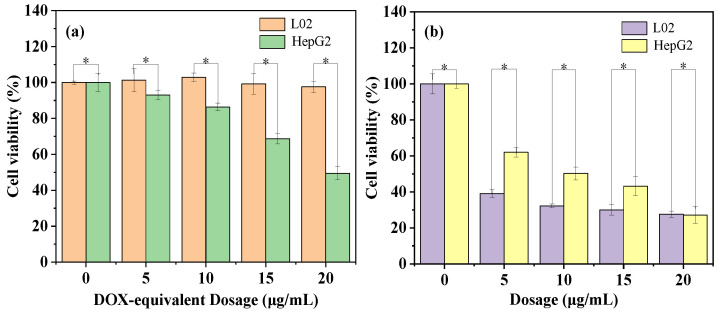
Cell viability assay in L02 and HepG2 cells of PDOX nanoparticles (**a**) and DOX (**b**) with different concentrations for 48 h, respectively. Values are expressed as mean ± SD (*n* = 6) (* denotes significant difference *p* < 0.05).

Finally, the cellular toxicity of the proposed PDOX nanoparticles was evaluated with the HepG2 cells and normal human liver cells (L02), in comparison with free DOX. The proposed PDOX nanoparticles exhibited a dosage-dependent cytotoxicity on the HepG2 cells but an excellent cytocompatibility on the L02 cells with a high cellular viability of 98%, even at a high DOX-equivalent dosage of 20 μg/mL (Figure 7a), while the free DOX gave higher cytotoxicity on the L02 cells (Figure 7b). Such differences may be caused by the redox responsiveness of the diselenide conjugation in the proposed PDOX nanoparticles, which showed different sensitivities to the GSH and ROS levels in normal cells and tumor cells. The results demonstrated the promising potential as a safe and efficient long-acting DSDS for tumor treatment.

At the same DOX-equivalent dosage, the cellular viability after incubation with the PDOX nanoparticles was higher than that with free DOX. This was caused by the slow drug release from the polyprodrug, as well as the lower antitumor efficacy of the released drugs, in which the amino group on DOX was derivatized, affecting its insertion in DNA [33]. However, the drug release could be accelerated with irradiation [20,21,22,23,24], providing a strategy to achieve a fast drug release if needed. So, individualized chemotherapy is expected with the proposed PDOX nanoparticles, by endowing both a faster drug release in the early stages of chemotherapy with exogenous irradiation, and slower drug release in the later stages for the recurrence of tumors.

## 3. Discussion

The proposed diselenide-containing polyprodrug PDOX showed a pH/redox co-triggered degradation and drug release owing to the alternate acid-labile acylhydrazone conjugation and redox-cleavable diselenide conjugation between the drug structural units in the main chain of the polyprodrug. Despite excellent stability in the normal physiological medium, such as blood circulation, as well as the intracellular microenvironment in normal cells, very slow drug release was achieved in the tumor intracellular microenvironment, similar to previous reports [34,35]. Such solubility-controlled drug release behavior was caused by the pH/reduction co-triggered DOX-SeH release and the pH/oxidation co-triggered DOX-SeOOH release. Despite the higher redox-responsive sensitivity of the diselenide bond compared to the disulfide one, such release drug derivatives hardly transfer into the parent drug DOX in the microenvironment, unlike the disulfide [36]. Thus, slower drug release and lower cytotoxicity resulted due to the lower solubility of the drug derivatives, DOX-SeH and DOX-SeOOH. Based on the results in the present work and other reported works [34,35], such combinations of amide/diselenide bonds may not be a better option for redox-triggered polyprodrugs. High antitumor efficacy is expected by designing diselenide-containing polyprodrugs using other combinations, which could release the parent drug after the redox cleavage of the disulfide bond.

## 4. Materials and Methods

### 4.1. Materials and Reagents

Selenium (Se, ≥99.99%), 4-chlorobutyryl chloride (98%), 1,8-diazabicyclo [5.4.0]undec-7-ene (DBU, 99%) and glutathione (GSH, 97%) were purchased from Shanghai Aladdin Bio-Chem Technology Co., Ltd. (Shanghai, China). Sodium borohydride (NaBH_4_, 96%) was purchased from Sinopharm Chemical Reagent Co., Ltd., Shanghai, China. Hydrogen peroxide (H_2_O_2_, 30%) was purchased from Damao Chemical Reagent Factory (Tianjin, China). Doxorubicin hydrochloride (DOX·HCl, 99.4%) was bought from Beijing Huafeng United Technology Co., Ltd. (Beijing, China). All other reagents and solvents were analytical grade and used directly as received. Deionized water was used throughout the experiments.

### 4.2. Analysis and Characterization

^1^H NMR measurements were conducted on a JNM-ECS 400 M instrument (JEOL, Tokyo, Japan). The relative molecular weight and polydispersity of the polyprodrug were measured on a gel permeation chromatograph (GPC) equipped with a Waters 1515 pump and a Waters 2414 differential refractive index detector, using DMF as the eluent at 35 °C. The morphology and particle size of the PDOX nanoparticles were observed on a JEM-2100 transmission electron microscope (TEM) (JEOL, Tokyo, Japan), sampling with their aqueous dispersions. Their hydrodynamic diameter and distribution were measured using dynamic light scattering (DLS, BI-200SM) in aqueous dispersion and the normalized scattering intensity was presented. The UV–vis spectra and drug content were detected using a TU-1901 UV–vis spectrometer (Beijing Purkinje General Instrument Co., Ltd., Beijing, China) at room temperature.

### 4.3. Synthesis Procedure

The pH/redox co-triggered degradable polyprodrug (PDOX) was synthesized via a facile one-pot two-step approach, including the open-ring reaction of selenolactone with the amino groups on D-DOX_ADH_ to form the intermediate (selenol derivative of D-DOX_ADH_) and the oxidation coupling reaction between the intermediates (Scheme 1).

Typically, D-DOX_ADH_ (70.0 mg, 0.0537 mmol, 1.0 eq) and triethylamine (TEA, 13.0 mg 0.129 mmol, 2.4 eq) were dissolved in 10 mL DMF. After adding selenolactone (48.1 mg, 0.323 mmol, 6.0 eq) and DBU (2.47 mg, 0.0162 mmol), the mixture was stirred in the dark at 40 °C for 72 h. The PDOX was collected by precipitation with ethanol and purified by three cycles of dissolving in DMF and precipitating with ethanol and dried in vacuum. (Yield: 38.19%); (^1^H NMR (400 MHz, DMSO-*d*_6_): δ = 10.49–10.20 ppm, 0.84H, H_a_, δ = 3.97–3.72 ppm, 4.95H, H_b+c_, δ = 1.13–1.01 ppm, 3.00H, H_e_).

### 4.4. Redox-Triggered Drug Release

The PDOX nanoparticles (1.0 mg) were dispersed in 10 mL of different release media, respectively. Then, the dispersions were transferred into dialysis bags (MWCO = 1000) and immersed in 100 mL of the corresponding buffer solution in an IS-RSD3 incubation shaker at 37 °C. At certain time intervals, 5.0 mL of the dialysate was taken out to measure the DOX concentration on a TU-1901 UV–vis spectrometer at 480 nm and 5.0 mL of the fresh buffer solution was added to maintain constant volume. The experiments were carried out in triplicate and the data presented are the average of three measurements.

### 4.5. In Vitro Cellular Experiments

The cellular uptake of the PDOX nanoparticles and subcellular distribution of the released drugs were visualized through fluorescence microscope (OLYMPUS, IX71), after incubating HepG2 cells with a PDOX dosage of 15 μg/mL for 48 h. Briefly, the cell nucleus was stained with 4′,6-diamidino-2-phenylindole (DAPI), and the fluorescent images were obtained at 405 nm for DAPI and 480 nm for DOX, respectively.

The MTT assay was used to evaluate the cytotoxicity of the PDOX nanoparticles and free DOX against the HepG2 and L02 cells (National Collection of Authenticated Cell Cultures). The cells were incubated in a 96-well plate with a concentration of 1 × 10^5^ per well at 37 °C for 48 h. Then, the PDOX nanoparticles or free DOX was added at different concentrations for a co-incubation of 48 h. After that, MTT (5.0 mg/L) was added to each well, followed by incubation for another 4 h. Finally, the cell viability was measured using the enzyme-linked immunosorbent assay appliance at 490 nm, after removing the crystals by dissolving in 150 μL of DMSO for 20 min. All the data are presented as the mean value of six measurements.

## 5. Conclusions

In summary, a novel safe and efficient long-acting tumor-specific DSDS was developed for future tumor treatment, by designing a polyprodrug with drug molecules as structural units in the polymer main chain, linked with acid-labile acylhydrazone and redox-cleavable diselenide conjugations. It could only be triggered to degrade and release drugs in acidic media with a high GSH or ROS level, like the tumor intracellular microenvironment. Such a feature makes it safe to normal cells but toxic to tumor cells. The drug release profiles indicated that the slow drug release was controlled by the solubility of the degraded products. Owing to the irradiation responsiveness of the diselenide bond, a faster drug release could be expected using exogenous irradiation, showing promising potential as an efficient individualized chemotherapy strategy for the different therapeutic stages in future tumor treatments.

## Data Availability

The data presented in this study are available in this article.
